# Atypical Presentation of Prostate Cancer and the Workup of an Adenocarcinoma of Unknown Primary

**DOI:** 10.4021/wjon482w

**Published:** 2012-08-26

**Authors:** Asim Ahmad, Winston Tan

**Affiliations:** a Internal Medicine Resident Mayo Clinic Florida, Jacksonville, Florida, USA; b Division of Hematology/Oncology Mayo Clinic Florida, Jacksonville, Florida, USA

**Keywords:** Prostate adenocarcinoma, Adenocarcinoma of unkown primary

## Abstract

Prostate Adenocarcinoma is one of the most commonly diagnosed cancers in the United States, with a prevalence of around 2.4 million. Patients with this disease commonly present with urinary frequency and hesitancy, nocturia, and dysuria secondary to tumor enlargement. We present the case of a 60-year-old man with multiple-site biopsy proven metastatic prostate cancer that presented with neither urological or bone related signs or symptoms. His findings were rather atypical; they included dyspnea, pancytopenia, nausea, and chills. We then detail how we narrowed our diagnosis through a systemic process of elimination, and review the general workup of an adenocarcinoma of unknown primary in a male patient.

## Introduction

Prostate cancer is the most common cancer in men, often diagnosed by an abnormal prostate on digital rectal examination and an elevated prostatic surface antigen (PSA). Here, we present the case of a 60-year-old male with an atypical presentation of aggressive metastatic prostate cancer with biopsy proven multiple sites involvement, including the prostate, lung, duodenum, colon, and bone.

## Case Report

A 60-year-old male presented to our Emergency Department with a chief complaint of worsening dyspnea over the past 2 days. Approximately 3 months earlier, he had been doing yard work at his home, and had noticed 3 ticks on his arm which he promptly removed. Over the next few days, patient developed productive cough, and mild fatigue. He was diagnosed at an outside facility with pneumonia, and placed on levofloxacin. Because of persistent symptoms, and history of tick exposure, patient underwent serological testing at an outside facility and was diagnosed with acute Lyme disease and ehrlichiosis. He was later prescribed doxycycline and rifampin.

Upon presentation, patient also described a history of nausea and vomiting, night sweats, chills, and a 15 pound weight loss over the past 3 months. It was noted that patient had significant pancytopenia (RBC 9.7, WBC 3, Plat 48), as well as elevated transaminases (AST 314 U/L and ALT 71 U/L) and alkaline phosphatase (469 U/L). Given his history, signs, and symptoms a clinical diagnosis of Ehrlichia infection was suspected.

**Figure 1:**
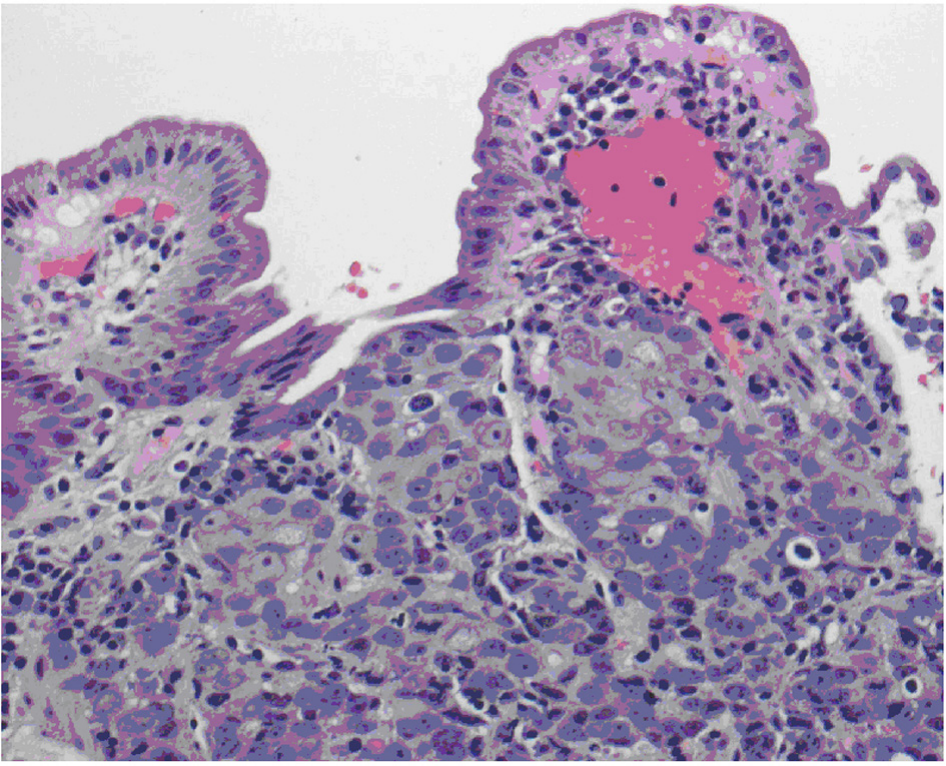
Biopsy of the duodenum demonstrating poorly differentiated adenocarcinoma, extensively infiltrating mucosa with ulceration of overlying epithelium. Extensive lymphovascular involvement is present.

Because of persistent dyspnea, a CT of the chest was ordered which was negative for pulmonary embolism in addition ground-glass opacities, nodules, and mediastinal lymphadenopathy. Endobrochial ultrasound (EBUS) and Bronchoalveolar lavage (BAL) were performed which were positive for malignancy, consistent with adenocarcinoma. Bone marrow biopsy was obtained to further assess the etiology of his pancytopenia, which showed diffuse bone marrow replacement by metastatic adenocarcinoma (Fig. 1). Cytokeratin, PSA, and CEA stains were all positive. CEA and PSA were elevated at 165 ng/mL and 132 ng/mL respectively. Patient stated last PSA was done 7 years ago was normal.

**Figure 2:**
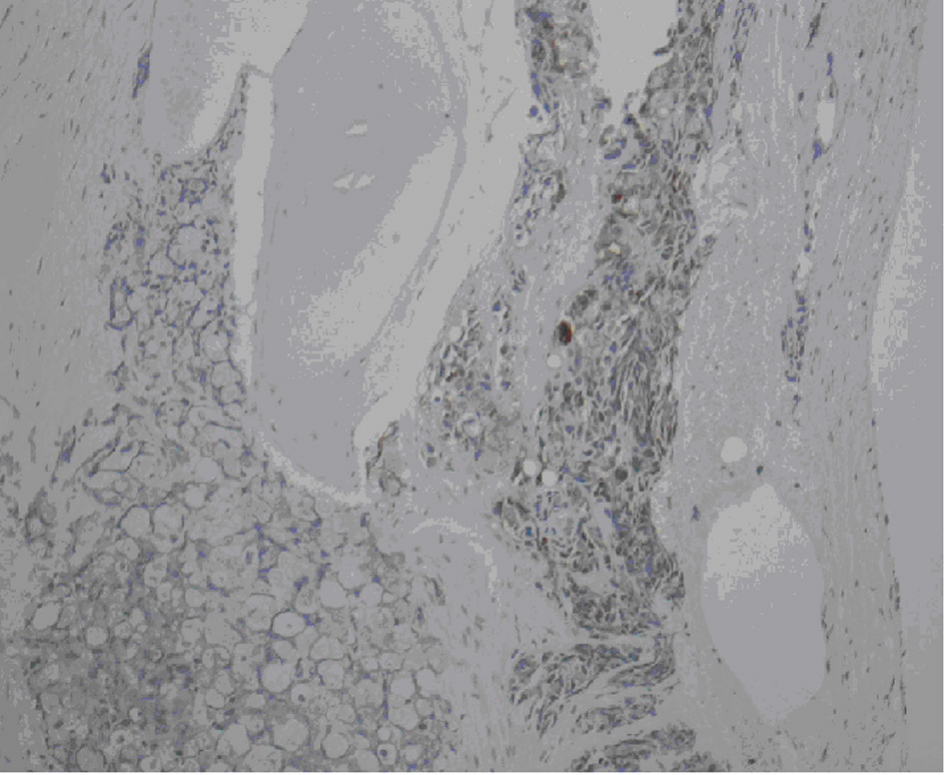
Bone marrow biopsy demonstrating diffuse marrow replacement by metastatic adenocarcinoma. Cellularity to fat ration 100:0. Erythropoiesis and granulopoiesis are decreased. Decreased number of lymphocytes and megakayocytes. Cytokeratin, PSA, and CEA stains are positive.

To assess his nausea and vomiting an upper endoscopy and colonoscopy were performed. Biopsies of suspicious lesions were obtained, these demonstrated adenocarcinoma of the colon and duodenum that was infiltrating the mucosa and vascular spaces. PSA stain was consistent with tumor cells from prostatic origin (Fig. 2). CT of abdomen showed extensive lymphadenopathy as well as liver lesions suspicious of metastases.

Prostate exam revealed nodularity, he stated last rectal exam was 3 years prior which was normal. Because of a palpable prostatic mass, a transrectal ultrasound-guided needle biopsy of the prostate was done which demonstrated adenocarcinoma present diffusely throughout the prostate. MRI of pelvis showed prostate malignancy with seminal vesicle invasion, pelvic lymphadenopathy, and diffuse skeletal metastasis. Bone scan was done which showed uptake throughout axial skeleton, ribs, and calvarium consistent with metastatic disease.

He was then started on bicalutamide, and after two weeks of therapy leuprolide was added. Additionally, zolendronic acid intravenously, calcium, and vitamin D were started. He had significant improvement of symptoms over the next 2 weeks.

## Discussion

Prostate adenocarcinoma is the most commonly diagnosed non-skin cancer in the United States of America [1]. The diagnosis is often suspected in a patient with an elevated PSA. Because prostate adenocarcinoma begins in the periphery of the gland and moves centrally, the classic findings of difficulty in voiding, dysuria, and increased urinary frequency, all secondary to urethral obstruction, occur relatively late in the disease.

The case we present here is unique for a number of reasons. From an epidemiological standpoint, this patient was diagnosed relatively young. The median age of diagnosis is 67; only 35% of males are diagnosed between the ages of 55 and 63. Likewise, the median age of death from prostate cancer is 80; 20% of patients die between the ages of 55 and 64, implying this patient had a particularly aggressive form of metastatic prostate cancer. Also given the size of the primary, one would have expected the patient to present with symptoms of urethral obstruction. This patient did present with other signs and symptoms secondary to metastasis. Dyspnea because of lung metastasis, nausea and vomiting because of colon and duodenum involvement, elevated LFTs secondary to liver metastasis and pancytopenia because of bone marrow replacement by metastatic adenocarcinoma.

Though bone, lung, and liver are the most common sites of metastasis, the way bone was involved in this case is fairly unique [2]. The most common sequela of metastases to the bone is initially osteoblastic [3]. The suppression of blood cell lines causing a pancytopenia is less common and generally a later finding in the disease course when compared to osteoblastic/osteoclastic activity. The mechanism is not fully understood, though recent studies in mice have demonstrated that prostate cancer cells can be directed to hematopoietic stem cell niches in bone marrow [4]. There, cancer cells have been shown to drive the terminal differentiation of hematopoietic stem cells, and thus cause a pancytopenia.

This atypical presentation alone would make diagnosing prostate adenocarcinoma difficult. This patient’s case was further complicated by a number of confounders. Patient’s history of tick exposure had raised concerns of an ehrlichia infection which may have explained the majority of this patient’s symptoms. Patient’s use of rifampin could have also explained some of these symptoms. The patient had a significant social history of having worked in Arizona; raising the possibility his symptoms were secondary to coccidioidomycosis infection. These would prove to be red herrings. After biopsy of lung lymph nodes, AUP became a likely diagnosis.

Carcinoma of unknown primary (CUP) refers to cancer in a patient in which after thorough medical workup with imaging, laboratory testing, and biopsy the primary site of the cancer remains unknown. AUP is a major subset of CUP, comprising 60-70% of all CUPs. In men, the most common causes of AUP are prostate, lung, pancreas, and colorectal cancer [5]. A significant proportion of cases in women involve breast cancer.

Workup of an unknown primary begins with a thorough history and physical. In this case, the history of present illness didn’t offer much that would help guide the clinician towards the correct primary site of disease; the signs and symptoms the patient presented with were secondary to metastatic foci. This is common as the patient’s chief complaint will often stem from the complications of the metastatic disease and not the primary itself. The history should focus on any previous lesions, any past history of biopsies, a complete family history to ascertain the risk of an inherited cancer syndrome, alcohol and smoking history, and a complete review of symptoms to help elucidate the etiology of the primary site. Interestingly, a complete 12 point review of symptoms failed to reveal any urinary symptoms that would lead the clinician to suspect adenocarcinoma of the prostate.

A complete physical exam can help guide the clinician in terms of a diagnostic workup. Special emphasis should be placed on assessing the lymph node groups, as well as a prostate exam in males. As part of a complete examination, a prostate exam was done which demonstrated nodularity in the 7 o’ clock position.

Based on patient’s pulmonary symptoms, a chest x-ray and CTA were obtained. CTA demonstrated lymphadenopathy, thought to be secondary to ehrlichiosis infection

Tissue samples were obtained from a subcarinal lymph node and a pretracheal lymph node conglomerate to rule out malignancy. This represents the next step in working up carcinoma of unknown primary, obtaining a pathological specimen from an easily accessible site that can undergo pathological analysis. Based on specimens provided, an initial diagnosis of adenocarcinoma of the lung was given to this patient. BAL was also performed and was positive for adenocarcinoma.

Once adenocarcinoma has been identified, immunohistochemical markers may be utilized to help determine the lineage of the tumor. This patient presented with pancytopenia, so a bone marrow biopsy was obtained. Similar to findings in the lung, this biopsy demonstrated adenocarcinoma. Cytokeartin, PSA, CEA, and P63 were all positive. It is important to understand the pattern of these markers and to correlate them with the clinical scenario in order to truly maximize their efficacy.

Based on history and physical, as well as analysis of initial pathological specimens, further biological, imaging, and endoscopic studies may be obtained. CT of patient’s abdomen revealed extensive lymphadenopathy and hypodense liver lesions suspicious for metastases. No pancreatic lesion was identified. Because of his GI symptoms, an esophagogastroduodenoscopy and colonoscopy were obtained. Biopsies demonstrated poorly differentiated adenocarcinoma infiltrating the mucosa and lymphovascular space in the colon and duodenum. Immunostains were positive for PSA.

Tumor markers by themselves are notoriously nonspecific; they have not demonstrated any benefit in establishing a primary site of a cancer and should not be ordered as a part of a nondirected diagnostic workup. However in this clinical case, where imaging, immunostains, and pathological specimens all pointed to a narrow differential, tumor markers were obtained to further help in the diagnosis.

To further support the diagnosis of prostate adenocarcinoma in this patient, a pelvic MRI was obtained. This demonstrated a large tumor in the posterolateral and posterior left base of the prostate, with extension into the left seminal vesicles. Significant extracapsular extention of the tumor was noted as well as diffuse skeletal metastases. Based on these findings, alongside the nodularity felt during digital rectal exam, a transrectal ultrasound-guided needle biopsy of the prostate was done which demonstrated adenocarcinoma present diffusely throughout the prostate with a Gleason score of 4 + 5 = 9.

To summarize; the workup of AUP begins with a complete history and physical. Though the history has not been shown to elucidate the primary site in the majority of patients, it does help point to areas where metastatic foci are likely. In this patient, a genitourinary exam including digital rectal exam helped in leading to the final diagnosis of prostatic adenocarcinoma, emphasizing the importance of a thorough physical exam. Biopsies of different sites were obtained to clarify whether the patient had a single malignancy or multiple malignancies at the same time. Imaging studies were obtained to help determine the sites of cancer involvement and identify the cause of his symptoms. CTs and MRIs are the imaging studies of choice, because of their superior diagnostic yield when compared to x-rays [6, 7]. To further study the tumor lineage, immunohistochemical stains are used.

This is a case of an aggressive prostate cancer with an atypical presentation that highlights the multiple sites of prostate metastasis. It demonstrates how with a thorough diagnostic approach the site of primary can be elucidated. The diagnosis leads to a specific treatment approach that leads to better palliation of patients with AUP.
